# From Staining Techniques to Artificial Intelligence: A Review of Colorectal Polyps Characterization

**DOI:** 10.3390/medicina60010089

**Published:** 2024-01-03

**Authors:** Kareem Khalaf, Mary Raina Angeli Fujiyoshi, Marco Spadaccini, Tommy Rizkala, Daryl Ramai, Matteo Colombo, Alessandro Fugazza, Antonio Facciorusso, Silvia Carrara, Cesare Hassan, Alessandro Repici

**Affiliations:** 1 Division of Gastroenterology, St. Michael’s Hospital, University of Toronto, Toronto, ON M5B 1T8, Canada; kareem.khalaf@mail.utoronto.ca (K.K.); raina.abadmd@gmail.com (M.R.A.F.); 2 Digestive Diseases Center, Showa University Koto Toyosu Hospital, Tokyo 135-8577, Japan; 3 Department of Endoscopy, Humanitas Research Hospital, IRCCS, 20089 Rozzano, Italy; tommyrizkala@outlook.com (T.R.); matteo.colombo@humanitas.it (M.C.); alessandro.fugazza@humanitas.it (A.F.); silvia.carrara@humanitas.it (S.C.); cesareh@hotmail.com (C.H.); alessandro.repici@hunimed.eu (A.R.); 4 Department of Biomedical Sciences, Humanitas University, 20089 Rozzano, Italy; 5 Gastroenterology and Hepatology, University of Utah Health, Salt Lake City, UT 84132, USA; daryl.ramai@hsc.utah.edu; 6 Department of Endoscopy, Section of Gastroenterology, Department of Medical and Surgical Sciences, University of Foggia, 71122 Foggia, Italy; antonio.facciorusso@unifg.it

**Keywords:** image-enhanced endoscopy, artificial intelligence, chromoendoscopy, optical diagnosis, colorectal cancer

## Abstract

This review article provides a comprehensive overview of the evolving techniques in image-enhanced endoscopy (IEE) for the characterization of colorectal polyps, and the potential of artificial intelligence (AI) in revolutionizing the diagnostic accuracy of endoscopy. We discuss the historical use of dye-spray and virtual chromoendoscopy for the characterization of colorectal polyps, which are now being replaced with more advanced technologies. Specifically, we focus on the application of AI to create a “virtual biopsy” for the detection and characterization of colorectal polyps, with potential for replacing histopathological diagnosis. The incorporation of AI has the potential to provide an evolutionary learning system that aids in the diagnosis and management of patients with the best possible outcomes. A detailed analysis of the literature supporting AI-assisted diagnostic techniques for the detection and characterization of colorectal polyps, with a particular emphasis on AI’s characterization mechanism, is provided. The benefits of AI over traditional IEE techniques, including the reduction in human error in diagnosis, and its potential to provide an accurate diagnosis with similar accuracy to the gold standard are presented. However, the need for large-scale testing of AI in clinical practice and the importance of integrating patient data into the diagnostic process are acknowledged. In conclusion, the constant evolution of IEE technology and the potential for AI to revolutionize the field of endoscopy in the future are presented.

## 1. Introduction

Since the conception of the endoscopic discipline, continuous and evolving equipment has been essential to meet the need to improve the quality of screening programs [1]. With colorectal cancer currently situated as the second most lethal cancer and third in incidence, colorectal cancer screening programs need to be of the highest clinical value to meet patients’ needs [2]. To address this, novel image-enhanced endoscopy advances are continuously evolving to revolutionize the characterization of lesions in the lower gastrointestinal (GI) tract.

Historically, image-enhanced endoscopy (IEE) has played a distinctive role in the histologic characterization of colorectal polyps and prediction of the invasion depth of colorectal cancers [3]. For many decades, all screening colonoscopies followed a dissect or removal-of-lesion directive, due to the assumption that all lesions were precursors to colorectal cancer. Nowadays, as outlined by the European Society of Gastrointestinal Endoscopy (ESGE), specific characterization measures need to be in place to classify the directive to follow [4]. Furthermore, the ‘sessile serrated’ nomenclature has emerged, defining a histological feature of an aberrant proliferation center that differs from hyperplastic lesions in the foci of proliferation, both in the crypt bases and irregular points along the crypt lumen [3]. Sessile serrated lesions have been shown to contribute to a third of colorectal cancers and detailed classification systems have been developed in response [5].

Despite the added benefit of reducing time-consuming biopsies, pathological histology, being the reference standard for differentiating between polyp subtypes, has some degree of misdiagnosis owing to errors in the sampling or retrieval of specimens. A 10% error rate in the pathological discrimination between polyp subtypes can be assumed unless an enhanced reference standard is used. Ultimately, in clinical practice, optical diagnosis through endoscopy and pathology through biopsy may have similar accuracy on a per-polyp-detected basis [6].

It is vital to delineate, in terms of classifying lesions in the colon, the crucial understanding of small or diminutive lesions and large lesions and what the operator’s role is. Following the ESGE’s guidelines, adapted from the American Society for Gastrointestinal Endoscopy (ASGE), strategies involving the decision to resect, discard, or leave in situ are highly dependent on proper classification techniques that can accurately satisfy the operator’s decision. Following high consensual agreement, the ESGE suggests that ‘virtual chromoendoscopy’ and dye-based chromoendoscopy can be used, under strictly controlled conditions, for real-time optical diagnosis of diminutive (≤5 mm) or small colorectal polyps [4,7]. This directive’s purpose addresses the need to replace histopathological diagnosis but keep a high-quality characterization standard. The switch from tissue-biopsy-type diagnosis to optical should be reported in scale systems that are validated with photo evidence for referral under experienced supervision.

The predictive value of a positive diagnosis (PV) to distinguish adenoma vs. non-adenoma is of the essence in high-efficiency screening systems. The ability to determine a lesion from an optical diagnosis with high accuracy replaces an older ‘notion’ of what histopathological tissue biopsy is, not simply a time saving strategy but one with environmental and financial implications. Unfortunately, a lack of confidence in image-enhanced endoscopic technologies means that practitioners are still far from forsaking old methods and replacing them with new.

We provide a comprehensive review of the various different IEE techniques, from its conception with dye-spray staining chromoendoscopy to the use of virtual electronic technologies and the consequent development of different classification systems, and conclude with future technologies and artificial intelligence, as a revolutionary modality on the horizon.

## 2. Dye-Spray Chromoendoscopy

Dye-spray chromoendoscopy is the application of a stain onto the surface of interest, constituting an imperative pillar in IEE. The application of a dye improves the visualization of microstructures and vascular patterns of the lesions being investigated on mucosal surfaces [8]. Dye-spray chromoendoscopy is important in the screening of malignant and premalignant lesions, and to enhance diagnostic accuracy and the adequate determination of tissue biopsies. It is worth noting that dye chromoendoscopy is limited in its application due to the absence of standardized methods for the analysis of lesion identification and classification [9]. Studies have noted that although the use of stains in chromoendoscopy enhances tissue resolution, a more accurate classification system is required for higher accuracy in diagnosis. Dye-spray chromoendoscopy stains are classified depending on their mechanism of action: whether the use of absorptive, contrast, or reactive stains are warranted. Firstly, absorptive stains are selectively absorbed intracellularly; one example of this is the use of methylene blue in lower GI colonoscopies [1]. Secondly, contrast staining, being the most used type of stain, highlights the surface of lesions by accumulating in pits and troughs between cells; an example is the indigo carmine stain. Lastly, reactive staining uses stains that change color based on a chemical reaction [10].

## 3. Methylene Blue

Methylene blue was first used by Kida et al. to enhance the diagnostic method of detecting early malignant lesions of the stomach [10]. Today, methylene blue’s application is limited to absorption into the cells of the small intestine, and although not absorbed into the cells of the stomach and esophagus, it may be used as a contrast agent to reveal the surface profile of lesions [1].

The per-oral preparation of 200 mg is eight tablets of MB-MMX administered with a 4-liter polyethylene glycol-based bowel preparation. In IEE, the dye stains the colorectal mucosa resulting in contrast enhancement and easier detection. Methylene blue’s clinical purpose is the detection of colorectal neoplasms [11,12].

## 4. Indigo Carmine

Initially, indigo carmine was used in the past to assess kidney function [1]. Today, its application differs, in that it attenuates the topography of the mucosal surface. Indigo carmine is useful to differentiate between benign and malignant lesions and for facilitating the analysis of the surface structure of the lesion [13]. Furthermore, indigo carmine delineates the boundaries of an early-stage malignant lesion and estimates the depth of cancer invasion into the mucosa, which is important for the accurate determination of the correct treatment, as outlined in later sections [1].

The mode of solution is a 0.1–0.4% solution for a 0.2% dilution, mix 5 mL of 0.8% solution with 15 mL of sterile water. The mechanism of image-enhanced endoscopy revolves around the pooling of the dye in mucosal crevices with no cellular staining for the detection and diagnosis of colorectal neoplasm [11].

A main staple in the classification of lesions in the lower GI is the use of the pit pattern characterization that has been standardized with the use of a magnifying colonoscopy following a system known as Kudo’s classification [8]. Kudo’s classification is characterized by six groups that constitute different pit pattern types. Type 1 has pits of normal, round regular mucosa in size and arrangement. Type 2 has star-shaped or onion-shaped hyperplastic polyps in a regular arrangement. Type 3 is divided into two subtypes, I and S: type 3I shows tubular elongated pits, reflecting tubular adenoma with low-grade dysplasia; while type 3S is similar to type 3I but is more compact and smaller, indicating high-grade dysplasia. Type 4 has branched pits of villous adenomas. Type 5 is classified as a cancerous lesion and is further divided into two subtypes: type 5I, which shows irregular structure and can be seen in invasive and non-invasive cancers; and type 5N, which is non-structural atypia, suggests deep submucosal cancers. Kudo’s classification is useful in assessing the histological structures of a lesion, types 1 and 2 are non-neoplastic lesions, while types 3 and 4 are classified as adenomatous lesions [14].

It is worth noting that the Kudo classification system, which has an important role in defining crypt classification, is involved in characterizing lesions in almost all other types of classification systems: virtual and dye-spray chromoendoscopy.

## 5. Virtual Chromoendoscopy

Virtual chromoendoscopy directly captures electronic signals analyzed by various image processing technologies. IEE is one such technology that converts color- and structure-based diagnostic information into a quantitative value that indicates its preprogrammed objective. In comparison to dye-spray chromoendoscopy, virtual chromoendoscopy is important in the elimination of time-consuming procedures such as stain spraying and suctioning. Multiple different modalities of this technology exist, such as narrow-band imaging, blue laser imaging, linked color imaging, I-scan, and FICE, all outlined below.

## 6. Narrow-Band Imaging (NBI)

Narrow-band imaging (NBI) revolutionized IEE, especially with the development of classification systems that allowed other IEE systems to classify lesions in the lower GI tract. NBI provides a method of optical chromoendoscopy, providing optical biopsies that enable diagnosis though a visual assessment, bypassing the need to perform a tissue biopsy. This modality in combination with magnification endoscopy improves the clarity and accuracy of visually based assessments of mucosal microvasculature and microstructure [15]. NBI can delineate pitting patterns of polyps with higher accuracy, enabling the clinician to differentiate between adenomatous polyps which require removal and inflammatory or hyperplastic polyps which do not need to be removed [16]. NBI visualization of the microvascular network to differentiate between neoplastic and non-neoplastic lesions was first described by Machida et al. [17]; later, the use of vessel thickness detected by NBI to assess the histological grade and depth of invasion of colorectal tumors was proposed by Hirata et al. [18]. Consequently, multiple classification systems developed to enhance this IEE modality advanced both the lesion detection and classification.

An optical digital IEE was developed by Olympus Medical Systems with an inbuilt mechanism for the modification of light sources with narrowed wavelengths to enhance both surface and capillary patterns for the detection and diagnosis of colorectal neoplasm. Previous equipment used for IEE utilized conventional red–green–blue light filters that offer a red hue on the projection, which made detection measures difficult [11]. Comparatively NBI allows light frequencies of 415–540 nm. Frequencies at 415 nm, constituting blue light, match the absorption spectrum of hemoglobin, which consequently highlights the capillaries in the superficial mucosa, and frequencies at 540 nm, green light, penetrate deeper into the mucosa. NBI provides greater clarity of mucosal surface structures and better visualization of mucosal vasculature networks [1].

The basic principle of NBI is that the depth of light penetration into the tissues is proportional to the wavelength used: blue bandwidth for superficial tissues, green bandwidth for intermediate tissues, and red bandwidth for deep tissues. In newer NBI systems, the combination of an upgraded light source and an additional blue light filter provides the scope to achieve near focus and a high-resolution macro function, producing brighter and clearer images that can detect lesions and discern fine details of the mucosa, especially the mucosal micro-surface and microvascular patterns [19].

Studies exploring NBI technology of still images have shown poor representation in accurate polyp characterization. Conversely, studies in a video-simulated clip managed to reach a diagnostic accuracy of 81% vs. 93% for experts (*p* < 0.05) in characterizing adenomatous polyps, providing a suitable modality for supplementing optical diagnostic decisions to endoscopists.

### 6.1. Sano Classification

Sano et al., in 2009 [20], described capillary patterns of variable lesions, studying microvascular architecture with the use of NBI magnification. Sano’s classification is divided into three types: type 1 is a hyperplastic pathology, showing a honeycomb pattern around a mucosal gland. Type 2 are lesions of the microscopic type that appear dark brown with near-vascular lesions that are easily identifiable using NBI colonoscopy without magnification. Type 3 are lesions defined as showing an irregular unstructured pattern in a mesh-like microvasculature, showing a regular size with branching and or irregular pattern. Type three is subdivided into type 3A and type 3B: type 3A lesions show a clearly visible microvasculature with a high microvascular density that lacks uniformity, has branching, and is irregular. Type 3B lesions show clearly visible demarcation between normal and cancerous mucosa on the surface based on the presence of a nearly avascular or a loose microvascular area.

### 6.2. NBI International Colorectal Endoscopic (NICE) Classification

The NICE classification was proposed in 2009 as a simple international classification for colorectal tumors. The NICE classification is based on the vessel color and surface pattern of the colorectal tumors observed in non-magnifying endoscopy. Studies have shown the usefulness in diagnosing colorectal polyp histology and predicting the deep submucosal invasion of carcinomas [21,22]. The NICE classification is divided into three types; type one shows the same or lighter color than the background that surrounds the growth, and no vessels, or isolated lacy vessels that are present coursing across the lesion [23]. Furthermore, there is the existence of dark or white spots of uniform size, or a homogeneous absence of a pattern on the surface after growth. Type 2 shows a brown color of the growth arising from vessels that appear as surrounding white structures with an oval, tubular, or branched white structure surrounding the brown vessels on the surface pattern of the growth. Type 3 has a brown to dark color in comparison, with the background being a patchy white area, where vessels shown are markedly distorted and may be missing and show diffuse areas of distortion or the absence of a pattern on the surface of the growth. Type one indicates a hyperplastic pathology, while type 2 indicates an adenomatous pathology, and finally, type 3 likely indicates deep submucosal invasive cancer. It is important to note that due to both the lesion color and vessel thickness being subjective estimates in the Kudo pit pattern classification, a system (which is the NICE classification) that validates this estimation was developed.

### 6.3. Japan NBI Expert Team Classification

The Japan NBI expert team classification was proposed in 2014 when several NBI experts reached a consensus to establish a universal NBI magnifying endoscope classification. The Japan NBI classification has three types. Similar to the NICE classification, each type is dependent on the vessel pattern, surface pattern, and the most likely pathology [19]. Type one has an invisible vessel pattern with regular dark or white spots, similar to the surrounding normal mucosa. On the surface pattern, it is most likely indicative of a hyperplastic polyp or a sessile serrated polyp. Type 2A has a vessel pattern on a regular caliber, with a regular distribution of a meshed or spiral pattern, the surface is regular and could be tubular, branched, or papillary, indicating a pathology of a low-grade intramucosal neoplasia. The type 2B vessel pattern is of variable caliber with an irregular distribution in its surface pattern. The pattern is irregular or obscure and indicates a high-grade intramucosal neoplasia or shallow submucosal invasive cancer. Type 3 shows a loose vessel area with interruption of thick vessels on the surface pattern. This amorphous area indicates a deep submucosal invasive cancer pathology [21].

### 6.4. WASP or the Workgroup on Serrated Polyps and Polyposis Classification

The workgroup on serrated polyps and polyposis classification or WASP is an extension of the NICE classification. Its core revolves around the recognition of sessile serrated polyps as precursor lesions of colorectal cancers, something dismissed in previous classification systems [24]. WASP offers the analysis of features such as an indistinct border, cloud-like surface, irregular shape, and dark spots inside the crypts. The diagnosis of a serrated sessile polyp is considered sufficient if two of the features mentioned above are present. One study, that studied the accuracy of the optical diagnosis of serrated sessile polyps following the WASP classification, found a high confidence, with 84% accuracy and a negative predictive value of 91% [24].

### 6.5. Unified Magnifying Endoscopic Classification (UMEC)

Recently, Inoue et al. [25] developed a new optical diagnosis classification which unified the criteria for the esophagus, stomach, and colon. This diagnostic classification is called the Unified Magnifying Endoscopic Classification (UMEC) [25]. It divides optical diagnosis into non-neoplastic, intramucosal neoplasia, and deep submucosal invasive cancer. This new diagnostic rubric was created to provide non-expert endoscopists and general gastroenterologists with a simple diagnostic criterion common to the GI tract. It does not aim to replace existing organ-specific classifications used by expert endoscopists. For the colon, UMEC basically adapted the JNET classification. UMEC 1 represents hyperplastic or sessile serrated polyps, UMEC 2A low-grade dysplasia, UMEC 2B high-grade dysplasia or shallow submucosal invasive cancer, and UMEC 3 deep submucosal invasive cancer. In the feasibility pilot study performed in 2021, the overall diagnostic accuracy for colorectal adenocarcinomas using the UMEC was 93.3%.

## 7. Flexible Spectral Imaging Color Enhancement (FICE)

FICE or flexible spectral imaging color enhancement is a proprietary digital imaging post-processing system developed by Fujinon. Recent advances in image processing technology have made it possible to estimate the specific wavelengths of light forming images, a process known as spectral estimation technology [21]. FICE uses a wide range of spectral combinations, termed multiband imaging, which enhances the mucosal surfaces and microcirculation. This is possible due to the selection of wavelengths in the visible light spectrum detected by humans, which is in the frequency range of 400 to 700 nm. FICE exploits this by producing 60 types of spectral images in the visible light range at 5 nm intervals, thus producing 300 types of image and theoretically creating 2700 × 10^4^ images from one endoscopic image [26].

The currently available research on five devices indicates the superiority of FICE images over white light images in terms of their resolution adaptability to different tissues. In 2009, Teixiera et al. described a classification system for FICE based on the magnified microvessel pattern types. Types one and two showed few, short, straight, and sparsely distributed vessels. While types three, four, and five showed numerous, elongated, and torturous capillaries that were irregularly distributed [27]. A study showed that this classification provides good diagnostic accuracy for colonic polyps [28]. Another study that applied the NICE classification for NBI using FICE to differentiate adenomas from hyperplastic polyps showed an accuracy of only 77%, suggesting that classification systems may not be interchangeable between advanced imaging modalities such as FICE [29]. Conversely, there has not been any established tissue diagnosis or differentiation capability capabilities with FICE technologies. The FICE system has not been shown to produce high-contrast images of the microvasculature of the mucosal surface [21].

## 8. Blue Laser Imaging (BLI)

The blue laser imaging system developed by Fujifilm was developed to address the problem of the limited availability of technologies that produce high-contrast images of the microvasculature on mucosal surfaces. BLI was the first endoscopic system to use a laser as the light source which allows the system to produce white light images as well as narrow-band images based on the properties of laser light. In comparison to the conventional Xe lamp used in NBI, the laser light source also has a lower power consumption and extremely low heat generation [30,31].

BLI uses an optical digital image inbuilt mechanism that modifies a light source with laser light and narrowed wavelengths to enhance capillary patterns for the detection and diagnosis of colorectal neoplasms [11]. This is achieved by allowing the light source that is best suited for the target tissue to be selected by adjusting the ratio of the two laser intensities of the white-light-laser and BLI modes. The white-light-mode laser produces a wide-spectrum source unsuitable for normal observation, with a shortwave narrow-band laser light source producing a narrow spectrum to highlight the microvasculature of the mucosal surface, thus identifying subtle changes in the mucosal surface that provide key information for the characterization of lesions [1].

For the consideration of morphological features, crypt, and vessel features of polyps, in addition to the plethora of existing classification systems aforementioned, the BLI adenoma serrated international classification or BASIC classification was developed. In recent studies, the BASIC classification was examined in a clinical setting in live patients and was found to have a high negative predictive value for sessile serrated polyps. In another study, endoscopy experts identified, with the use of the BASIC system, conventional adenoma (vs. non-adenoma) with 94.4% accuracy, 95% sensitivity, 93.8% specificity, 93.8% positive predictive value, and 94.9% negative predictive value. Moreover, the study validated the use of the BASIC classification system to characterize colorectal polyps with >90% accuracy and a high level of interobserver agreement [32].

## 9. Linked Color Imaging (LCI)

Linked color imaging was primarily developed on the mechanistic basis of the BLI modality. The idea stems from the use of a Xe lamp source that, although it provides a brighter image, remains darker than white light, which results in a lower accurate-diagnostic rate [33]. LCI is an optical digital IEE modality developed by Fujifilm with a mechanism that utilizes the application of pre- and post-processing software technology to enhance color pattern or structure for the detection of colorectal neoplasms [11]. The utilization of additional red wavelengths, in comparison to BLI, allows LCI to produce images of sufficient brightness that are similar to images produced by white light, enhancing the red color spectrum, thus allowing lesions to be identified easily. The red color spectrum is important for the detection of colorectal polyps and adenomas, for example, the visualization of a red color in a splash or purple color under LCI [8]. LCI has been regarded as being primarily used for the detection of lesions instead of its characterization property.

## 10. I-Scan

I-scan is a software-based digital post-processing image-enhanced technology. I-scan performs three functions: surface enhancement, contrast enhancement, and tone enhancement. Similar to dye-spray chromoendoscopy’s use of acetic acid, which highlights changes in the mucosal surface by forming a blue hue on darker regions, I-scan identifies and examines lesions by obtaining luminance intensity data from each pixel, outlining surface enhancements based on the light–dark contrast [34]. Additionally, tone enhancement divides the image based on normal white light into its RGB components, converting a resynthesized image. The produced images have a subtle hue difference which highlights vascular patterns or subtle changes in the mucosa [21]. Bouwens et al. developed the I-scan classification for endoscopic diagnosis (ICE) based on the Kudo and NICE classifications. The ICE classification considers color, epithelial surface pattern, and vascular pattern. The studies then evaluated images and found 79% sensitivity, 86% specificity, and 81% accuracy for the ICE classification system [35]. Unfortunately, only a small number of studies have investigated the usability of I-scan systems [21], and the studies that have been performed provide conflicting data. Two specific randomized prospective studies contradict each other, with the first reporting that high-definition colonoscopy with I-scan is better than standard video colonoscopy in detecting colorectal neoplastic lesions. Ironically, the other demonstrated that I-scan did not improve the ADR and failed to prevent missed polyps when compared to high-definition colonoscopy [21].

## 11. Endocytoscopy (EC)

Endocytoscopy is an ultra-high magnification endoscopic technique developed for in vivo assessment of GI tract lesions. This technique involves the use of intraprocedural stains to allow microscopic visualization of the mucosal surface [36]. The first-generation endocytoscope was developed in 2003 and has since been enhanced several times, leading to the latest fourth-generation endocytoscopes (GIF-H290EC; Olympus Medical Systems Corp., Tokyo, Japan), which provide up to 500x continuous zoom-focus magnification.

Based on the available literature, three different staining methods have been reported when using EC: toluidine blue, methylene blue, and crystal violet. In 2010, the CM double-staining method was developed and has been utilized in subsequent studies since this staining method produces a staining pattern that resembles the traditional hematoxylin-eosin staining in conventional microscopy [37]. The CM double-staining method consists of 10cc of 0.05% crystal violet and 1cc of 1% methylene blue.

For colorectal lesions, Kudo et al. developed a novel EC classification in 2011 [38] which focuses on structural and cellular atypia such as lumen morphology and nuclear changes. In this classification, EC1a and EC1b are diagnosed as non-neoplastic, while EC2, EC3a, and EC3b are diagnosed as neoplastic. An RCT in 2013 [39] showed a 94% diagnostic accuracy for colorectal neoplasms. A study in 2018 [40] also assessed whether EC can be used to accurately determine the histologic grade of T1 colorectal cancer. This study reported a 91.5% diagnostic accuracy for well-differentiated colorectal adenocarcinomas, and 91.5% for moderately differentiated colorectal adenocarcinomas.

## 12. Clinical Functions of IEE

### 12.1. The Efficacy of a Characterization IEE System Is Measured by Its Ability in the Detection of Colorectal Polyps, Accurate Polyp Characterization. For Some Specific Techniques It Can also Be Used for the Prediction of the Depth of Invasion into Mucosal Surfaces

The adenoma detection rate (ADR) is the most widely used colonoscopy quality indicator, an increase in the ADR is associated with a decrease in post-colonoscopy CRC. A colonoscopy is a user-dependent procedure and endoscopists have variable adenoma detection rates [8]. The evaluation of detection rates is not the scope of this review; however, technologies such as dye-spray and virtual chromoendoscopy and artificial intelligence have the potential to increase adenoma and polyp detection rates in screening routine colonoscopies. A meta-analysis from Shinozaki et al. showed a 26% percent increase in the ADR when linked color imaging was used in comparison with white light imaging. Moreover, in recent years, a large number of studies have investigated the effect of artificial intelligence on the ADR. A recent meta-analysis from Hassan et al. that analyzed 21 randomized control trials, comparing standard colonoscopy versus colonoscopy with the use Computer Aided Detection (CADe) systems (computer-aided detection), showed a 24% increase in the ADR [41]. This study proved the advantages of using this form of colonoscopy, though detection rates are not in the scope of this review.

### 12.2. Characterization

Most of the important classification and characterization systems in dye-spray and virtual chromoendoscopy are mentioned above. Moreover, computer-aided diagnosis systems (CADx) are trained to differentiate between adenoma or non-adenoma polyps. The majority of polyps encountered during colonoscopy are diminutive polyps (≤5 mm), these polyps have a very low malignant potential; moreover, when present in the rectosigmoid colon these polyps do not require removal [42]. The latest guidelines from the ESGE and ASGE have proposed two optical diagnosis strategies: the resect-and-discard strategy and the leave-in-situ strategy [4,43]. The resect-and-discard strategy intends to resect a polyp that has been diagnosed, to remove it but not to send it to pathology. On the other hand, the leave-in-situ strategy intends to leave in place a polyp that was detected as this polyp is believed not to have a malignant potential. The evaluation of each of these strategies is not in the scope of this review, we decided to present them in a simplified way.

According to the ESGE, the leave-in-situ strategy can only be implemented on diminutive polyps (≤5 mm) that were diagnosed by the endoscopist with high confidence on their part [4]. Moreover, the optical diagnosis strategy used should have at least 80% sensitivity and specificity diagnostic performance when compared to the gold standard pathology diagnosis [4].

For the leave-in-situ strategy, the ESGE restricts the strategy to only diminutive polyps in the rectosigmoid colon that were also diagnosed with high confidence by the endoscopist. And the diagnostic performance threshold for the implementation of the leave-in-situ strategy is at least 90% sensitivity and 80% specificity, when compared to the gold standard pathology diagnosis [4]. Both strategies can save on the cost of colonoscopy by decreasing the number of materials used for the removal of polyps and by decreasing the material used for pathological reports. However, harm can occur if a wrong optical biopsy is made.

CADx systems have shown the most promising results in assisting endoscopists for the optical biopsy of diminutive polyps [30]. In fact, most studies have proved that the CADx standalone performance during real-time colonoscopy has matched the threshold of the current guidelines.

### 12.3. Prediction

Although out of the scope of our review, IEE can help predict the invasion depth of colorectal cancer. The decision to perform minimally invasive surgery or endoscopic submucosal dissection (ESD) is based on an accurate prediction of the depth of invasion [44]. Whether this is dye-spray chromoendoscopy, with the use of indigo carmine to identify neoplastic invasive pattern Kudo type 5–1 with a demarcated area of type 5N, or the observation of the capillary vasculature and surface pattern, any of the criteria for virtual chromoendoscopy will implement a strategy for the management of these lesions [45]. In summary, a three-step strategy is outlined, with the first step being a conventional colonoscopy for the identification of the lesion, and the second step being narrow-band virtual chromoendoscopy to delineate the capillary pattern. Capillary pattern type 1 requires a follow-up, while type 2 and type 3A having a demarcated area requiring endoscopic resection. For capillary types 3B and 3A with a positive surface pattern or negative surface pattern, a surgical intervention is highly recommended. The third step requires a dye-assisted chromoendoscopy for capillary pattern types 3B and 3A, with a demarcated area of a positive surface pattern [8]. Moreover, Kudo classification V1, indicating a non-invasive pathology, would undergo endoscopic resection, while V1 invasive morphology and VN would undergo a minimally invasive surgical resection [14]. Table 1 summarizes the different characterization criteria for deep submucosal invasion [19,23,45,46,47].

## 13. Artificial Intelligence (AI)

A common misconception regarding AI’s characterization mechanism is that it follows the methods that have been previously mentioned. It is worth noting that dye-spray and virtual chromoendoscopy utilize characterization based on the human eye. The beauty of AI is that its characterization standards are not based on human error, the systems use inherent algorithms learnt through prior computed patterns to discern a lesion’s character.

In accordance with the ESGE, recommendations for the possible incorporation of computer-aided diagnosis for the detection and characterization of lesions in colonoscopy should be implemented [49]. The guidelines state that high-quality multicenter in vivo clinical studies should be implemented and show acceptable and reproducible accuracy for colorectal neoplasia characterization. Although several points need to be addressed on the existing AI technology, its application for the characterization of colorectal polyps shows promise.

Endoscopic AI establishes an ‘optical biopsy’ through a computer-aided diagnosis (CAD) [50]. Theoretically identified lesions are defined as either to be diagnosed and discarded, resected and discarded, or resected and sent to pathology. There are multiple different programmed systems that have AI computer-based systems, but the basic principle of the technology is based on the addition of a computer program that identifies lesions with the help of previously established virtual chromoendoscopy modalities. Such modalities, like magnifying NBI, in addition to a CAD system, have aided endoscopists to differentiate between hyperplastic and adenomatous polyps. Initially, Tischendort et al. [51], in 2010, used images from a support vector machine to create a CAD classification model based on the microvasculature pattern and found 91.9% accuracy in characterizing 209 colorectal polyps, 76% of which were neoplastic. The overall study had 90% sensitivity, 70% specificity, and 85% accuracy in determining adenomatous vs. non-adenomatous polyps. In retrospect, an abundance of studies emerged in the following decade, with a heightened focus on NBI and white light (WL) instruments. In reference to these studies, one can follow Table 1, that being said, other studies on add-on modalities such as BLI, endocytoscopy, and laser-induced fluorescence spectroscopy technologies exist. Chronologically, by publication date, Gross et al. [52], in 2011, utilized a support vector machine in a prospective design magnifying NBI technology to compare small vs. non-adenomatous polyps, achieving an accuracy of 93% (95% and 90% for sensitivity and specificity, respectively). The importance of AI technology in characterizing small lesions and defining ‘adenomatous’ vs. ‘non-adenomatous’ revolutionizes our directive technique in factors that may aid us, the ‘operators’, in following resect-and-discard or leave-in-situ strategies.

A year later, Takemura et al. [48] revolutionarily used a convolutional neural network with magnifying NBI technology, achieving an accuracy of 98% (98% sensitivity and specificity). Convolutional neural networks that exploit patterns achieved an overall higher characterization rate than before. Furthermore, multiple studies researching CNNs’ characterization power, conveyed through a comparison of adenomatous vs. non-adenomatous polyps in studies that used NBI or WLI technologies, gave a wide array of results with highly acceptable accuracy.

The true innovative advantage AI systems offer was later shown to be advantageous in comparing diminutive adenomatous and hyperplastic polyps. In 2019, Byrne et al. [53] conducted a retrospective study using CNN—NBI and achieved 94% accuracy in properly characterizing diminutive adenomas (98% and 83% for sensitivity and specificity). Interestingly, Jin et al. [54] found 87% accuracy with (83% and 91% for sensitivity and specificity) proving a lower characterization power than what was previously established. Regrettably, in 2017, Koemeda et al. [55], utilizing a CNN for both NBI and WLI in a retrospective design, achieved a lower accuracy for a characterization of only 75% when comparing adenomatous vs. non-adenomatous polyps.

In 2020, Song et al. [56] published data on using a CNN system with NBI that compared adenomatous polyps with sessile serrated polyps. The retrospective design provided an accuracy in correctly characterizing lesions of 82% (84% and 88% for sensitivity and specificity). Moreover, a study by Zachariah et al. [57] investigating a similar outcome achieved a 97% negative predictive value for adenomas among diminutive rectosigmoid polyps independent of the use of NBI or WLI.

One very interesting and important meta-analysis encompassed twenty-six articles which the authors stated to be of medium quality but with no publication bias; these were stratified to test the accuracy and precision of the AI-assisted classification of identified colorectal polyps on colonoscopy. The study found that an overall AI-assisted classification of colorectal polyps yielded a sensitivity of 0.92, while detailed information on AI’s use in diminutive polyps and magnifying endoscopy is summarized in Table 2. The study concluded that AI-assisted technology provides an indispensable step into the future of detecting and characterizing colorectal polyps, with similar accuracy to the gold standard.

Nonetheless, the integration of AI into clinical practice is not without drawbacks and encounters legal and billing issues. Currently, AI systems are not widely adopted in clinical practices and they serve as assisting tools to aid endoscopists in decision making. To facilitate the widespread implementation of AI in clinical practice, standardized guidelines are essential to instruct endoscopists on how to use these systems. It is crucial for endoscopists to thoroughly evaluate AI systems before their deployment, given the variability in the training of each model and its diagnostic performance. Moreover, data on AI should undergo further large-scale testing and not be limited to expert centers. Looking ahead, there is an essential factor that needs to be implemented in AI systems that did not exist in previous IEEs, and that is the integration of a patient’s history, laboratories, and other relevant data, to provide an evolutionary learning system that aids the diagnosis and management of patients with the best possible outcomes [58,59].

## 14. Conclusions

In conclusion, regardless of the IEE modality a universal characterization standard should be always met to implement any diagnostic strategy, whether it is resected and dissected or left in situ. In the case of leave-in-situ strategies, a minimum of 90% sensitivity and 80% specificity should be achieved for a high confidence endoscopic characterization of diminutive colorectal lesions [60]. On the other hand, for resect-and-discard strategies, a minimum of 80% sensitivity and 80% specificity should be achieved for a high confidence endoscopic characterization of diminutive colorectal lesions.

The question remains, where do colorectal lesion characterization methods lie in today’s practice and what is the future? Today, we are advancing with technology at a rate much faster than ever before. One important aspect is that we need to establish a high-quality measure for characterizing debilitating cancerous lesions to validate the high accuracy of point optical diagnosis. The futuristic innovation of AI has truly revolutionized endoscopy as an imperative preventative tool in medicine and is constantly evolving.

## Figures and Tables

**Table 1:** Submucosal classification of invasive colorectal lesions.

IEE	Modality	Classification System	Criteria	Reference
Dye-Based Chromoendoscopy	Methylene Blue	Kudo’s Pit Pattern	Type V1 and demarcated area or Type VN	[45]
Dye-Based Chromoendoscopy	Indigo Carmine	Kudo’s Pit Pattern	Type V1 and demarcated area or Type VN	[45]
Virtual Chromoendoscopy	NBI	Sano	Type IIIB	[46]
Virtual Chromoendoscopy	NBI	NICE	Type 3	[23]
Virtual Chromoendoscopy	NBI	Japan NBI Expert Team	Type 2B high-grade and Type 3	[19]
Virtual Chromoendoscopy	NBI	WASP	Two of indistinct border, cloudlike surface, irregular shape, and dark sports inside the crypts	[47]
Artificial Intelligence	CAD	Convolutional Neural Network	Convolutional Neural Network	[48]

**Table 2:** Important studies that utilized AI-assisted devices for the accurate classification of colorectal lesions.

IEE	Modality	Classification System	Criteria	Reference
Dye-Based Chromoendoscopy	Methylene Blue	Kudo’s Pit Pattern	Type V1 and demarcated area or Type VN	[45]
Dye-Based Chromoendoscopy	Indigo Carmine	Kudo’s Pit Pattern	Type V1 and demarcated area or Type VN	[45]
Virtual Chromoendoscopy	NBI	Sano	Type IIIB	[46]
Virtual Chromoendoscopy	NBI	NICE	Type 3	[23]
Virtual Chromoendoscopy	NBI	Japan NBI Expert Team	Type 2B high-grade and Type 3	[19]
Virtual Chromoendoscopy	NBI	WASP	Two of indistinct border, cloudlike surface, irregular shape, and dark sports inside the crypts	[47]
Artificial Intelligence	CAD	Convolutional Neural Network	Convolutional neural network	[48]

## Data Availability

Not applicable.

## Abbreviations

European Society of Gastrointestinal Endoscopy, ESGE; gastrointestinal, GI; image-enhanced endoscopy, IEE; narrow-band imaging, NBI; blue laser imaging, BLI; linked color imaging, LCI; NBI international colorectal endoscopic, NICE; workgroup on serrated polyps and polyposis, WASP; Unified Magnifying Endoscopic Classification, UMEC; I-scan classification for endoscopy, ICE; flexible spectral image in color enhancement, FICE; artificial intelligence, AI; endoscopic submucosal dissection, ESD; computer-aided diagnosis, CAD; randomized controlled trials, RCTs; positive diagnosis, PV; convolutional neural network, CNN.
